# Occurrence of Nasal Nosocomial Myiasis by Lucilia sericata (Diptera: Calliphoridae) In North of Iran

**Published:** 2012

**Authors:** MR Youssefi, MT Rahimi, Z Marhaba

**Affiliations:** 1 Dept. of Veterinary Parasitology, Islamic Azad University, Babol Branch, Iran; 2 Young Researchers Club, Islamic Azad University, Babol Branch, Iran

**Keywords:** *Lucilia*, Myiasis, Maggot, Hospital, Iran

## Abstract

We report a case of human nasal nosocomial myiasis in a 69-year-old rural man with numerous live maggots in nostrils. The patient was admitted in Emergency Ward due to dyspnea. After 72 hours the companion of patient discovered larva. The presence of the third instar larva indicated that the infestation was not more than three days. The collected instars were cleared, fixed and morphological studies then were carried out precisely and the larva was identified as *Lucilia sericata*.

## Introduction

Myiasis is a term given to infection caused by fly maggots which dwell in live humans and animals temporary. Various species of flies are able to be agents of myiasis and caused other forms of parasitism (1, 2). Entomogically myiasis is classified in several types: obligatory, facultative and accidental. Clinically it can be categorized as a result of body involvement area such as cutaneous, nasopharyngeal, ophthalmic, and urogenital (3). In nasopharyngeal myiasis, larva causes ocular and aural involvement and larva targets at the head cavities including nose, sinuses, ears and eyes. The first report of myiasis caused by *Lucilia sericata* was in 1826 by Magen who extracted maggots from eyes, mouth and paranasal sinuses of a hospital patient (4).*Lucilia* fly belongs to Calliphoridae family which is ectoparasites both in humans and animals. *Lucilia* fly is metallic green or copper green, 8-10 mm and feed on carrion, excrement and garbage (1, 3). The female lays eggs on meat, corps, carcasses and wound of human and animals. Service 1986 has reported that the requisite time for developmental stage of *Lucilia* is about 10-23 days. Between 8-12 h, the eggs change into a conical larva, and complete peritreme of posterior respiratory spiracles. After 4–8 days, larvae develop and drop on soil and after 6-14 days change in to adult flies (3). According to our survey, the present case is the first report of nosocomial myiasis caused by *Lucilia* in Iran.

## Case report

**Fig. 1:**
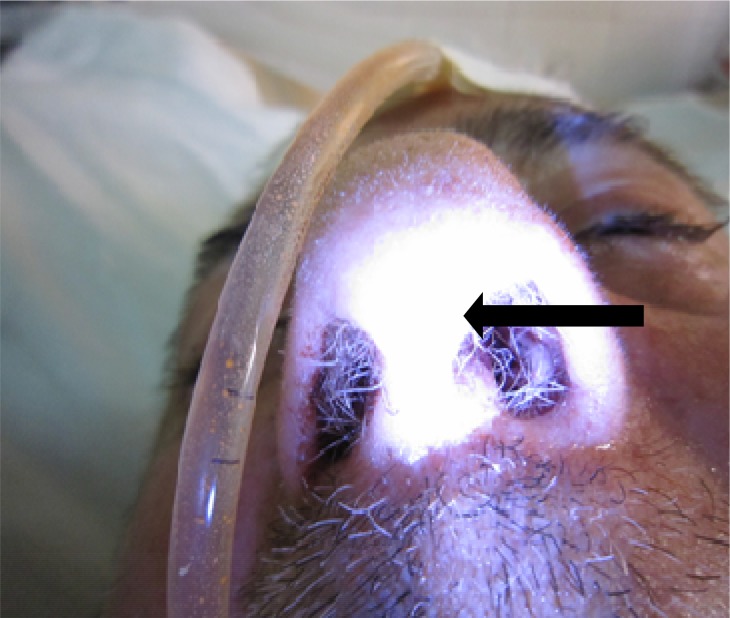
The arrow shows a *Lucilia* larva in left nostril of 69 year old man in hospital

The patient was 69 year old man resident in rural area of Babol, Mazandaran Province, northern Iran. Considering his major history points, he was a farmer that gave up smoking after 35 years. The patient was complaining about pulmonary problems for two years. Eventually, he was hospitalized in one of Babol hospitals, emergency ward. Signs and symptoms which pushed the patient to meet clinic at the time of submission in the hospital were dyspnea, stridor, coughing and nasal discharge. Furthermore, he was suffering from stomachache and ambiguous digestive problems. Overall, regarding vital signs, he had critical and serious conditions and he was in a semi-conscious state. The companion of patient unexpectedly observed some tiny, white wormlike living creatures which were motile and active in left nostril of him on the third day after admission (Fig. 1).The wormlike creatures were separated by a forceps and transferred to the Department of Parasitology, Babol Branch Islamic Azad University, for identification and examination. The number of extracted larva from the left nostril was considerably more than other side, nearly 50 to 7 and left nose shown some lesions, abscess and slight inflammation. The anterior of examined larva showed red color, probably because they fed on their host.

The third instar was preserved in 70% ethanol and cleared by lactophenol. After precise and comprehensive examination by aid of light microscope, the larva was identified *as L. sericata* according to the morphological characters of larva by using Walker identification key (5).

**Fig. 2:**
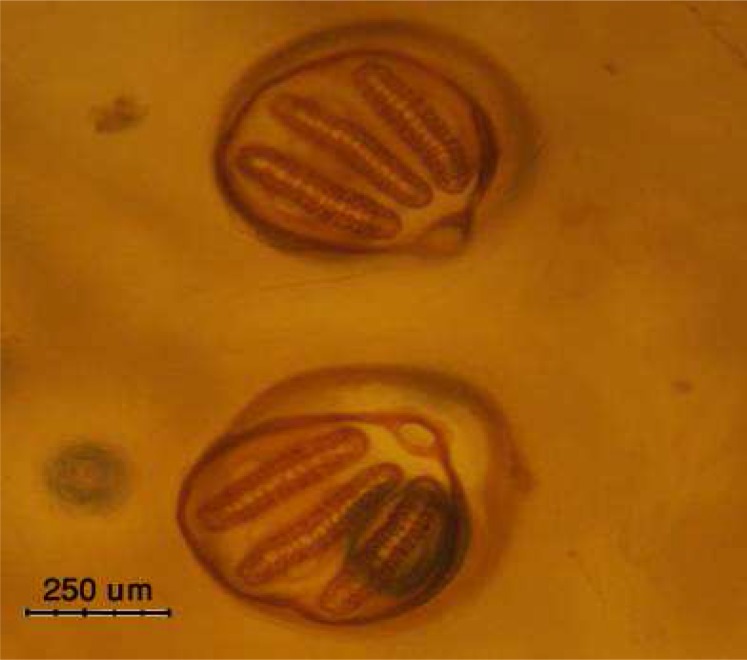
The posterior spiracle of *Lucilia sericata* instar

*Lucilia* larva can be differentiated from similar genes *Calliphora* by considering that larva body had tiny spines which arranged in distinct rows and the peritreme of posterior spiracle in *Lucilia* instar is narrow and it has just one internal spine while in *Calliphora* is thicker with two internal spines. In addition, the base of anterior spiracle in *Lucilia* is larger while in *Calliphora* is smaller. Moreover, it can be recognizable from *L. cuprina* instars which is an identical genus by considering that in anterior spiracle of *L. sericata* larva, 7-10 lobes can be observed in comparison with 4-5 lobes in *L. cuprina* which possesses a complete peritreme (Fig. 2)(5).

## Discussion

Nasal myiasis is caused by deposition of fly larva in the human nostrils. The occurrence of nasal myiasis in developing countries and tropical area is remarkable. Many species of *Diptera* can lead to nasal myiasis including: *Eristalis tenax, Sarcophaga* sp.*, Oestrus ovis, Phaenicia sericata, Chrysomya bezziana, Chochilomyia hominivorax* and *Drosophila melanogaster* in Iran, Turkey, Algeria, Arizona, Malaysia, French Guiana, Turkey, respectively (6–12). Nosocomial myiasis usually occurs in debilitated patients (13). The first nosocomial myiasis in Iran was reported by Minar in 1998 (14). Moreover, in Kuwait, nosocomial myiasis was occurred by *L. sericata* on the fourth and fifth days after admission. The patient was 10 years old boy who was hospitalized after accident due to head wound and comatose and maggots emerged from his nostril and ears (15).

A case of nasal myiasis was reported from a 70 years old woman by *L. sericata* in Korea (16). Edalat reported urogenital myiasis caused by *L. sericata* in an 86 years old rural man with penile ulcer from Iran (17). Talari reported a wound and auricular myiasis by *Lucilia* from a patient who was addicted to opium for 12 years and a 3-year history of heroin injections in Iran (18).

Surprisingly *Lucilia myasis* annually causes economic losses approximately $161million in Australia (19). Attempts to develop vaccines on livestock versus myiasis and fly attacks have been partially successful (20).

Considering the facts that separating of larva from nostrils of patient occurred 72 hours after admission to the clinic and by knowing the life cycle of *Lucilia* that the required time for eggs in order to change into conical larva, and completing peritreme of posterior respiratory spiracles is between 8-12 hours and after 4-8 days, larva develop and drop on to soil and after 6-14 days transform into adult flies, it can be concluded that nasal myiasis of the patient happened in the hospital.

Our report illustrates several interesting points. It accentuates prominent role of sanitation and hygiene in hospitals and rural area for the prevention of myiasis. Installing screens with small mesh size in front of hospital windows to exclude insects can be effective and efficient method for preventing from nosocomial myiasis. In addition, lack of awareness of patient was predisposing factor for larva infestation. Immobile, severely ill, semi-conscious and comatose patients are highly vulnerable groups to myiasis and nasal cavity is one of proper sites for this occurrence. It is also noteworthy that this is the second report of nasal myiasis in Iran and is the first report of this type of myiasis which caused by *Lucilia* in our country.
